# Direct Detection of *Candida albicans* with a Membrane Based Electrochemical Impedance Spectroscopy Sensor

**DOI:** 10.3390/s18072214

**Published:** 2018-07-10

**Authors:** Dorota Kwasny, Sheida Esmail Tehrani, Catarina Almeida, Ida Schjødt, Maria Dimaki, Winnie E. Svendsen

**Affiliations:** 1 Department of Micro- and Nanotechnology, Technical University of Denmark, rsteds Plads, Building 345 B, 2800 Kgs. Lyngby, Denmark; dorota.kwasny@nanotech.dtu.dk (D.K.); shesme@nanotech.dtu.dk (S.E.T.); cgs.almeida@gmail.com (C.A.); madi@nanotech.dtu.dk (M.D.); 2 Department of Haematology, Copenhagen University Hospital, Rigshospitalet, 2100 Copenhagen, Denmark; ida.schjoedt@regionh.dk

**Keywords:** Candida, electrochemical impedance spectroscopy, membrane based sensor

## Abstract

Candidemia and invasive candidiasis is a cause of high mortality and morbidity rates among hospitalized patients worldwide. The occurrence of the infections increases due to the complexity of the patients and overuse of the antifungal therapy. The current *Candida* detection method includes blood culturing which is a lengthy procedure and thus delays the administration of the antifungal therapy. Even though the results are available after 48 h it is still the gold standard in pathogen detection in a hospital setting. In this work we present an electrochemical impedance sensor that is capable of detecting *Candida albicans* yeast. The yeast cells are captured on electrodes specifically functionalized with anti-Candida antibodies and detection is achieved by electrochemical impedance spectroscopy. The sensor allows for detection of the yeast cells at clinically relevant concentrations in less than 1 h.

## 1. Introduction

Patients with impaired immunoprotection have a high risk of infection. The risk group is incredibly large, including cancer, HIV positive or post-transplant patients [1,2]. Infections that have a mild effect on a healthy individual can be fatal for many of these patients if the disease is not detected early enough. If infections are left untreated and allowed to progress into systematic diseases they are a significant cause of mortality among immunocompromised patients [3,4].

There are many pathogenic microorganisms such as virus, bacteria or fungi that can cause a disease in a population of patients with an impaired immune response. Fungal infections have recently become a major concern for the health of immunocompromised patients and their current diagnosis is lengthy and lacks accuracy [1,5]. A common cause of infections is fungi of *Candida species*, in particular *Candida albicans* (*C.albicans*). It is an opportunistic pathogen that causes severe illness among immunocompromised patients [2,4,5]. Candidiasis is mainly an endogenous infection produced by the overgrowth of fungi due to physiological changes in the body, e.g., the imbalances in the gut microbiota [3,6,7]. However, it can also be acquired from sources like catheters or prosthetic devices, by person-to-person or vertical transmission [1,3,6,8,9]. When the infection spreads to the bloodstream it is referred to as invasive candidiasis and can spread to other body organs [2]. Invasive candidiasis is the fourth most common cause of hospital-acquired infections and *Candida sp.* are responsible for 50% of diagnosed fungal bloodstream infections in US [1,2].

The gold standard of the current detection method is a blood culture [4,8,10]. It requires sterile collection of a patient’s blood sample, which is then cultured to test for fungal growth. The weakness of this method is primarily the time-to-answer, which takes at least 2 days but also its low sensitivity as various factors affect the growth of *C. albicans* in a laboratory setting [1]. Other diagnostic tests based on detection of *C. albicans* cell wall components, mainly polysaccharides, such as mannan and β-D-glucan are considered beneficial [4,5,8,9]. However, their diagnostic value is undermined by many described cases of false positive results, as their concentration in blood is not specific and can be affected by dietary requirements or former administration of drugs [5,8]. A lot of effort has been put in establishing a successful polymerase chain reaction (PCR) protocol for detection of *C. albicans* in a blood sample. Although fast and species specific, this method amplifies also DNA from non-viable *C. albicans* cells, leading to false positives [5,11]. Other diagnostic methods involving detection of host anti-*Candida* specific antibodies are a valid choice but their use in immunodeficient patients is limited [8,9]. Another measurable indication of a fungal infection is the ratio of D-/L-arabitol in urine, which has a strong diagnostic value among neonatal patients [12].

Some of the described tests have been miniaturized in a form of sensors to address the need for an improved diagnostic method for *C. albicans*. The selected method should provide fast diagnostics at low cost and with high sensitivity to detect clinically relevant concentrations at the levels of 10–100 colony forming unit (CFU) present in 1 mL of blood [6]. Similarly to the current gold standard of detection, some sensors aim for direct detection of *C. albicans* cells. One method to directly detect fungi is based on the mass detection with cantilever sensors [13]. A sensor based on field effect-transistor was able to detect *C. albicans* at 50 CFU/mL [6]. Other sensor, proposed by Mulero and others [14] is based on micropore technology, which measures electrical properties of the transmigration event of *C. albicans* at concentrations as low as 20 CFU/mL. A recent example of a photonic crystal sensor based on mannan recognition was proposed with detection limit of the order of 32 CFU/mL [15]. Even though a sensitive and fast detection is possible with mentioned sensors, their complex microfabrication influences the overall cost per test.

The objective of this study is to develop a simple sensor for specific detection of *C. albicans*. We present a membrane-based electrochemical sensor with a functional layer of anti-*C. albicans* antibodies able to specifically detect the presence of *C. albicans* spiked in PBS at a concentration as low as 10 CFU/mL in less than 1 h.

## 2. Materials and Methods

### 2.1. Membrane Electrodes Fabrication and Design

The electrochemical sensor used in this work is based on our previously developed membrane electrodes as described in [16,17,18]. Briefly, the shadow mask with the electrodes design was laser ablated in 0.5 mm thick polymethylmethacrylate (PMMA) using a CO${}_{2}$ laser (Epilog Laser, Golden, CO, USA). The polycarbonate (PC) membranes (Millipore) with 5 μm pore size were aligned manually underneath the shadow mask ensuring that the pattern with the electrodes was positioned above each membrane. Afterwards, a single step deposition of 100 nm of gold layer using E-beam evaporation was performed to create the gold electrode pattern on each membrane.

**Figure 1:**
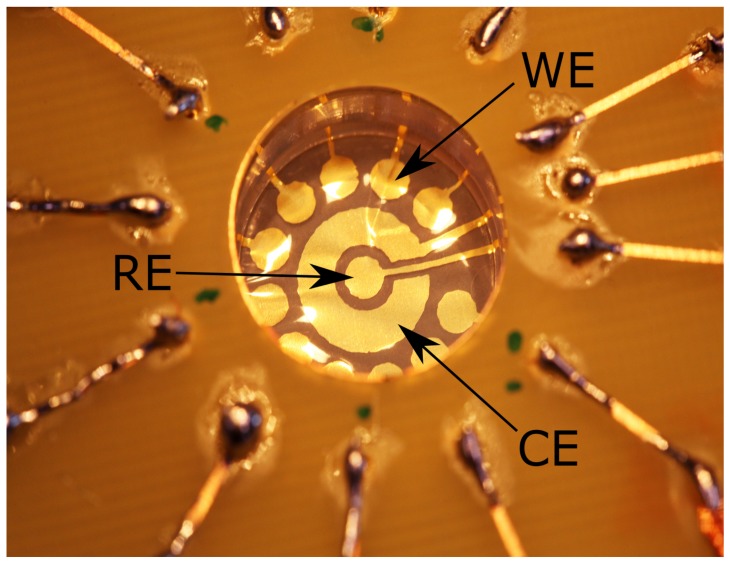
Final design of membrane electrodes enclosed in measurement holder. A central circular electrode serves as a pseudo-reference (RE), around it a large ring electrode is a counter electrode (CE). The RE and CE are surrounded by 11 working electrodes (WE) at the edge of the membrane.

The membrane electrodes chip shown in Figure 1 is composed of individually addressable electrodes for electrochemical measurements. The circular electrode in the middle is a reference electrode (RE), the large ring electrode in the middle is a counter electrode (CE) and the remaining 11 circular electrodes are working electrodes (WE). The membrane electrodes chip is placed in a custom made PMMA holder with a liquid chamber in the middle, sealed by a silicone o-ring. The electrical connections to the potentiostat are formed through a PCB with soldered spring pins and wires.

### 2.2. Membrane Electrodes Functionalization

The polyclonal anti-Candida albicans antibody were acquired from Abcam (ab53891, Cambridge, UK). Functionalization of the gold electrodes with antibodies was performed following a modified protocol described in [19]. The first step requires modification of the antibodies with a crosslinker by incubating 2 mg/mL antibody solution with freshly prepared 20 mM solution of sulfo-LC-SPDP (sulfosuccinimidyl 6-[3’(2-pyridyldithio)-propionamido]hexanoate) (Thermo Scientific, Waltham, MA, USA) for 1 h at room temperature. After the incubation the reaction by-products and unreacted sulfo-LC-SPDP are removed by means of desalting column (Zeba Spin, Thermo Scientific). Once the antibodies are modified with disulfides a 100 μg/mL solution is prepared and incubated overnight with the membrane electrodes.

### 2.3. Yeast Growth

*Candida albicans* (ATCC) stock solution was grown for 24–48 h at 37 ${}^{\circ }$C in a potato dextrose broth (Sigma Aldrich). After this time dilution series in PBS was prepared from 10${}^{-1}$ to 10${}^{-6}$. The dilutions of 10${}^{-4}$, 10${}^{-5}$ and 10${}^{-6}$ were plated on potato dextrose agar plates and grown overnight at 37 ${}^{\circ }$C before counting the colonies to determine the colony forming units present in each dilution sample. Afterwards, solutions of 10, 100 and 1000 CFU/mL were prepared based on the colonies count, by further diluting in PBS as necessary. The same procedure was applied for the growth of *Saccharomyces cerevisiae* (bakery yeast) that were used as a control.

### 2.4. Sensor Development and Sample Analysis

All sensor measurements were performed using an Autolab potentiostat (N series and multiautolab-2 channel-equipped with FRA module) in the frequency range of 100 kHz to 0.3 Hz at 10 mV AC voltage vs open circuit potential.

The initial measurement of the developed sensor was done after antibody immobilization in potassium hexacyanoferrate (II/III)(1:1) (Sigma Aldrich) at 10 mM in PBS to determine the baseline signal. Afterwards, control (*S. cerevisiae*) and test (*C. albicans*) samples at different concentrations were incubated on the electrodes for 15 min followed by 2 × 5 min washing in solution containing PBS + 0.01% Tween20 and 1 × 5 min in PBS. Subsequently, a solution containing a redox couple of potassium hexacyanoferrate (II/III)(1:1) at 10 mM in PBS was added and the electrochemical impedance spectroscopy was performed.

The sensor of the electrochemical system can be modelled with a modified Randles circuit, including a constant phase element and the charge transfer resistance ($R_{ct}$). The latter is used for measuring the changes upon surface modifications and target binding as it is sensitive towards changes occurring at the surface of the electrode. The Nova software (version 1.11, http://www.ecochemie.nl) was used for modelling the equivalent circuit using ’Fit and simulation’ analysis and the $R_{ct}$ values were determined using the ’Electrochemical circle fit’.

## 3. Results

### 3.1. Characterization of the Antibody-Functionalized Electrodes

**Figure 2:**
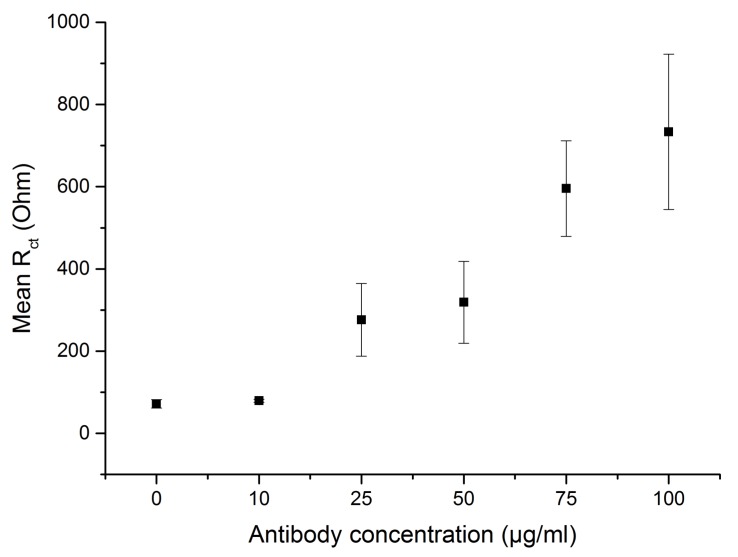
An EIS measurements to test the optimal antibody concentration (0, 10, 25, 50, 75 and 100 μg/mL) to cover the electrodes surface. The charge transfer resistance was extracted from the measured Nyquist plots and plotted against each antibody concentration. The highest charge transfer resistance was measured for 100 μg/mL and used for further experiments.

The anti-Candida antibodies used here were modified with the LC-SPDP molecule, which is a linker molecule that introduces disulfides to primary amines on antibodies. The addition of disulfide groups on the antibodies allows for their immobilization on gold electrodes. The attachment of antibodies at various concentrations was monitored electrochemically with impedance spectroscopy measurements. The charge transfer resistance of the antibody modified layer was analysed to determine the most appropriate concentration for immobilization (Figure 2).

In Figure 2 the change of the charge transfer resistance of the electrodes upon immobilization of the increasing concentration of antibodies is shown. At concentration of 75 μg/mL and above the antibodies are covering more of the electrodes surface, which is represented by a charge transfer resistance of 600 Ohms and above. At concentration 50 μg/mL and below a fast charge transfer is observed at a value of 300 Ohms. The flattening of the curve was not observed in the concentration range used here. The concentration of 100 μg/mL was selected for further measurements as a compromise between high charge transfer resistance (good antibody coverage) and keeping the cost of the device low, since antibodies are expensive.

### 3.2. Sensor Sensitivity and Specificity

To test the sensor sensitivity different concentrations of *C. albicans* solution in PBS were incubated on the functionalized membrane. The pre-functionalized membrane was prepared fresh prior to the measurements. The tested concentrations of *C. albicans* time was 15 min, followed by a thorough washing for 15 min. All measurements were performed in 10 mM potassium hexacyanoferrate (II/III)(1:1) solution in PBS and the impedance spectroscopy was performed.

**Figure 3:**
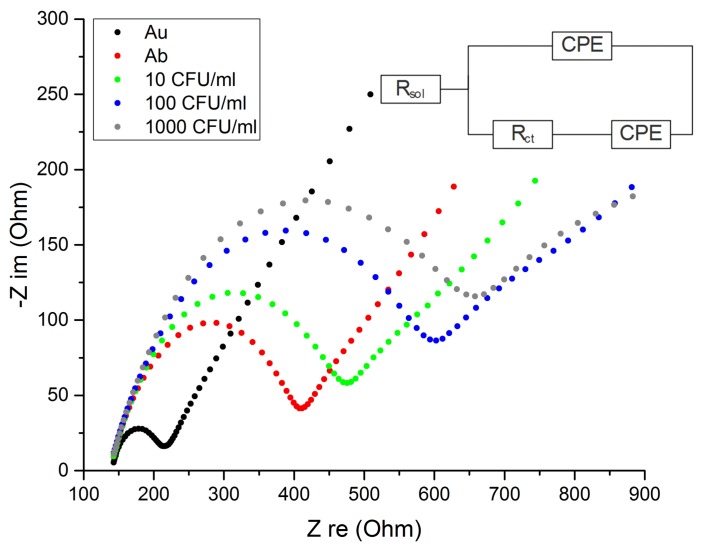
Nyquist plot for clean gold (Au-black), antibody modified (Ab-red) and each tested *C. albicans* concentration with 10, 100 and 100 CFU/mL (green, blue and grey respectively). The data is fitted to the modified Randles equivalent circuit.

The raw data in the form of a Nyquist plot are shown in Figure 3. Initially the electrode charge transfer resistance is low at around 77 Ohms and a significant increase up to 278 Ohms is observed after the immobilization of the antibodies. The incubation of the functionalized electrodes with increasing concentrations of *C. albicans* showed a further increase in $R_{ct}$ to 350, 500, 578 Ohms for 10, 100 and 1000 CFU/mL respectively.

Electrochemical impedance spectroscopy data were normalized to allow for sensor to sensor comparison. The following equation was used for the data normalization:

(1) $$\begin{array}{c} DeltaR{}_{ct}=\frac{R_{ct}-R_{ct0}}{R_{ct0}}\times 100\%. \end{array}$$

**Figure 4:**
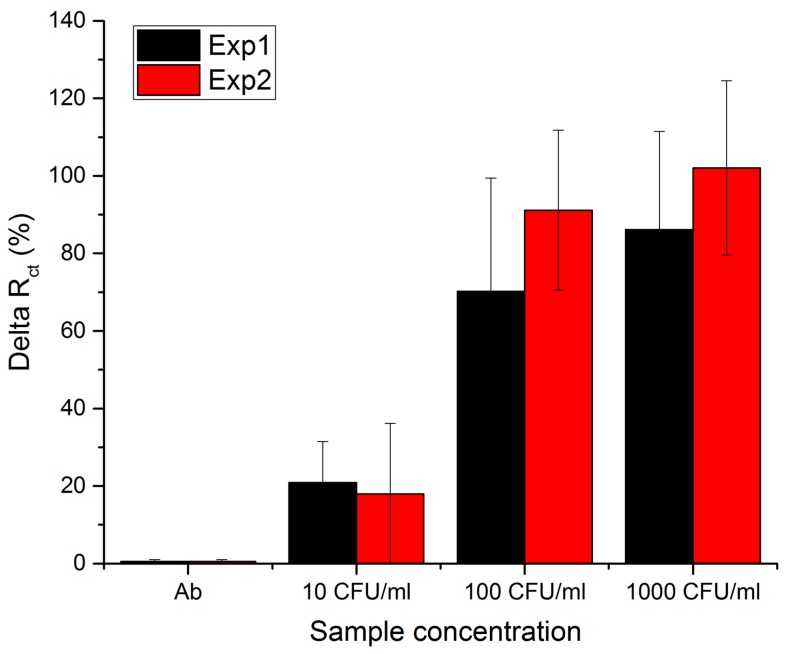
The bar graph of normalized charge transfer resistance ($DeltaR{}_{ct}$) for each tested *C. albicans* concentration with 10, 100 and 100 CFU/mL. The data comes from two independent experiments in which data from at least 4 electrodes was recorded. The increase in $R_{ct}$ value is attributed to binding of *C. albicans* to the antibodies on the electrode surface.

In Figure 4 the results are plotted as a change of charge transfer resistance ($DeltaR{}_{ct}$) in response to increasing amounts of *C. albicans* in the sample. The graph shows the charge transfer resistance in percentage relative to the antibody functionalized surface. It can be seen that after the sensor has been exposed to increasing concentrations of *C. albicans* sample, the charge transfer resistance of the electrodes increases. The experiment was repeated twice, using at least 4 individual working electrodes per membrane sensor in each experiment. The sensor responded with an increase in charge transfer resistance towards the lowest tested concentration of 10 CFU/mL with the observed change of around 20% relative to the charge transfer resistance of the antibody functionalized sensor. With increasing sample concentrations the sensor’s response also increases. The highest increase of almost 100% was observed for 1000 CFU/mL in the 2nd experiment.

The change of charge transfer resistance is different for each experiment. The starting point for each experiment was the average sensor’s impedance after immobilization of antibodies, which differed for each membrane. The normalization of the data to this baseline, eliminated the electrode-to-electrode variation, but not the membrane-to-membrane difference. Even though each sensor has a different response level the data are comparable and led to conclusion of the sensor’s sensitivity towards 10 CFU/mL.

**Figure 5:**
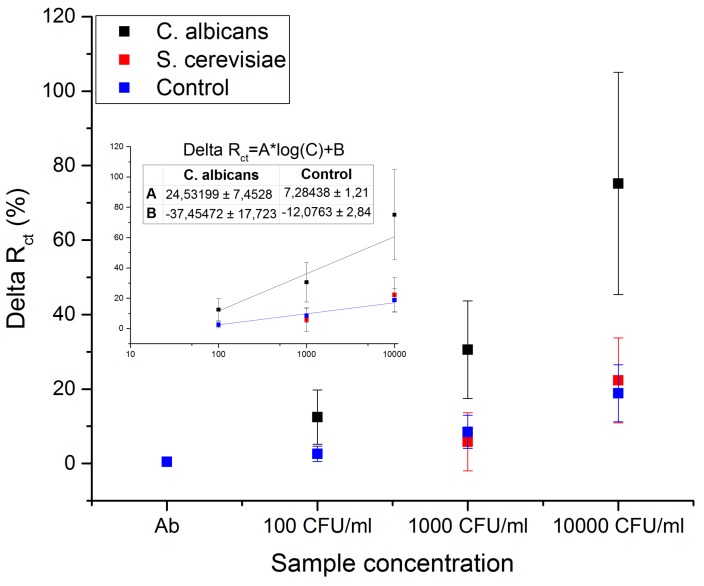
The specificity of the sensor is shown in the graph of normalized charge transfer resistance ($DeltaR{}_{ct}$) for *S. cerevisiae* and *C. albicans* on the sensor surface with or without antibodies. The data from 3 experiments are normalized and averaged. Only binding of *C. albicans* evoked a significant change in charge transfer resistance, whereas control and *S. cerevisiae* binding resulted in only small change in $R_{ct}$. The linear fit of the data is shown in the graph insert together with the equation of the calibration curve for *C. albicans* and control.

In order to evaluate the specificity, the sensor was exposed to increasing concentrations of *C. albicans* and *S. cerevisiae*. A non-functionalized membrane served as a negative control for investigating unspecific binding. The results are shown in Figure 5. The experiment was repeated 3 times, the data are shown as a percentage change of the charge transfer resistance relative to the antibody functionalized surface. The lowest tested concentration of *C. albicans* was 100 CFU/mL.

It can be seen that the charge transfer resistance of the sensor increases with higher concentrations of *C. albicans* in the tested sample (black squares in Figure 5). The change in charge transfer resistance as a function of *C. albicans* concentration has a linearity fit of $R^{2}=0.916$. The negative control (blue squares in Figure 5) resulted in a negligible increment in charge transfer resistance for the highest tested concentration of 1000 CFU/mL of *C. albicans*. The linearity fit for negative control is $R^{2}=0.973$ (Figure 5). Similarly to the negative control, incubation of *S. cerevisiae* (red squares in Figure 5) caused a small increase of the charge transfer resistance, at the level comparable to the negative control. It was attributed to unspecific absorption and accumulation of microorganisms on the hydrophobic surface of the sensor. No linear fit for *S. cerevisiae* is provided due to insufficient data, however, we suspect a similar trend to that of the negative control.

## 4. Discussion

By monitoring the change of charge transfer resistance of the electrodes we measure the binding between the antibody and *C. albicans*. The methods applied in this study is an electrochemical impedance spectroscopy which is known for its sensitivity towards changes occurring on a sensor surface. Our data presented in this study have demonstrated that a simple geometry of gold electrodes functionalized in a one-step process can successfully be applied for detection of *C. albicans*.

The functionalization of the electrodes is the most important step in the development of a functional biosensor. The biological recognition layer of antibodies needs to be stable over the period of measurements and highly reproducible. Each membrane sensor consisted of 11 working electrodes functionalized with antibodies through a self-assembly process. The nature of the process has induced some electrode-to-electrode variations, which can be eliminated by a use of more controlled functionalization protocol. Furthermore, the modification of the antibodies with a linker molecule can potentially affect their reactivity. An orientated immobilization of antibodies using Protein A assisted immobilization was tested, however, it was incompatible with the membrane material. Nevertheless, results achieved with standard electrochemical electrodes (gold on silicon oxide) show that orientated immobilization improves reproducibility. We are currently modifying the protocol to make it compatible with the membrane electrodes.

The antibody modification protocol used in this study ends after introduction of the disulfide groups. The standard protocol would involve further reduction of the disulfides to thiols as they have high affinity towards gold. However, due to high reactivity of thiols there is a risk of cluster formation between the molecules instead of attachment to gold. Furthermore, it has been known that disulfides have equally high affinity to gold as thiols [20] but are less reactive and thus chosen in this study for the overnight functionalization scheme. Moreover, the reducing agents, such as dithiotreithol often used in the procedure, destroy the disulfide bridges in the native form of the antibodies affecting their target binding capacity. The main advantage of the chosen protocol is the one-step immobilisation and ease of antibody modification. However, the addition of disulfide groups to primary amines can affect the orientation of the antibodies as the primary amines are present throughout the entire structure of the antibody.

We demonstrated a sensitivity of the biosensor down to 10 CFU/mL in a PBS sample, which is at the clinically relevant levels. The normalization of the results was done to accommodate the sensor-to-sensor variation due to the self-assembly process of antibodies immobilization. Each sensor contains 11 working electrodes, however for the lowest tested concentration of 10 CFU/mL, the resistance increased only for few tested electrodes. Hence, the large standard deviation. This can be explained by the fact that the volume of 100 μL used for incubation of *C. albicans* with the membrane sensor only contains a few *C. albicans* cells available for binding to the electrode surface. Considering the fact that the antibodies modified with disulfides bind to any gold surface on the membrane sensor some of the *C. albicans* present in such a low concentrated sample can easily bind to reference or counter electrodes and will not be detected on the working electrode.

We have shown that our sensor can successfully distinguish other yeast cells and its specificity will be further explored with other *Candida sp.* The sensing was performed in less than an hour including the washing steps that can be optimized in the future to shorten the time-to-answer even further. In the current study the porosity of the membrane is not used in the experiments; however, we envision using the porous structure for simultaneous filtering of the blood cells and sensing of the pathogens in the blood sample. If necessary, membranes with smaller pores will be used to avoid loss of *Candida* cells through the pores.

## 5. Conclusions

In this article we have shown a potential for electrochemical impedance detection of *Candida albicans* at clinically relevant concentrations. The developed sensor showed high sensitivity and specificity towards *Candida albicans* providing accurate and fast detection in less than 1 h. Future work requires optimization of the sensor functionalization protocol to ensure more reliable target binding. The membrane based sensor will be further modified to enable blood processing by filtration and diagnostics of *Candida albicans* infections directly from blood samples. When combined with the blood sample preparation, our sensor is a promising diagnostic tool for detecting *Candida* infections in immunocompromised patients which aids fast diagnostics to improve the treatment with antifungal drugs.
